# The effects of adjunctive treatment with l-carnitine on monitoring laboratory variables in ICU patients: a double-blinded randomized controlled clinical trial

**DOI:** 10.1186/s13063-022-07010-4

**Published:** 2023-01-03

**Authors:** Farveh Yahyapoor, Mahdi Keshani, Alireza Sedaghat, Awat Feizi, Cain C. T. Clark, Mohammad Bagherniya, Mohammad Safarian, Mohaddeseh Badpeyma, Abdolreza Norouzy

**Affiliations:** 1 Department of Nutrition, Faculty of Medicine, Mashhad University of Medical Sciences, Mashhad, Iran; 2 Food Security Research Center, Isfahan University of Medical Sciences, Isfahan, Iran; 3 Department of Community Nutrition, School of Nutrition and Food Science, Isfahan University of Medical Sciences, Isfahan, Iran; 4 Department of Anesthesiology, Faculty of Medicine, Mashhad University of Medical Sciences, Mashhad, Iran; 5 Biostatistics and Epidemiology Department, School of Health, Isfahan University of Medical Sciences, Isfahan, Iran; 6 Centre for Intelligent Healthcare, Coventry University, Coventry, CV1 5FB UK; 7 Anesthesia and Critical Care Research Center, Isfahan University of Medical Sciences, Isfahan, Iran; 8 Student Research Committee, Tabriz University of Medical Sciences, Tabriz, Iran; 9 Department of Nutrition, Faculty of Nutrition and Food Sciences, Tabriz University of Medical Sciences, Tabriz, Iran

**Keywords:** l-Carnitine, ICU, Nutritional status, Monitoring variable, Supplementation

## Abstract

**Background:**

Critically ill patients must be monitored constantly in intensive care units (ICUs). Among many laboratory variables, nutritional status indicators are a key role in the prognosis of diseases. We investigated the effects of l-carnitine adjunctive therapy on monitoring variables in critical illness.

**Method:**

A prospective, double-blind, randomized controlled trial was implemented in a medical ICU. Participants were 54 patients, aged > 18 years, with multiple conditions, randomly assigned to receive 3 g l-carnitine per day or placebo, along with enteral feeding, for 1 week. Primary outcomes included monitoring variables related to nutritional status.

**Result:**

Of 54 patients randomly assigned, 51 completed the trial. Serum albumin (Alb) (*P-*value: 0.001), total protein (*P*-value: 0.003), and calcium (Ca) (0.044) significantly increased in the intervention vs. control group. Alanine transaminase (ALT) (0.022), lactate (<0.001), creatinine (Cr) (0.005), and international normalized ratio (INR) (0.049) decreased meaningfully in the intervention vs. control group.

**Conclusion:**

l-Carnitine supplementation in critically ill patients can improve several parameters including INR, Cr, ALT, lactate, Ca, Alb, and total protein.

**Trial registration:**

Iranian Registry of Clinical Trials IRCT 20151108024938N2. This trial was approved by the Research Ethics Committee of Mashhad University of Medical Sciences (registration code: IR.MUMS.fm.REC.1396.671) (available at https://en.irct.ir/trial/30748, May 2018).

## Introduction: background and objectives

Patients who are admitted to the intensive care unit (ICU) are, typically, critically ill with one or more complications and comorbidities, such as multiple organ failure, acute pancreatitis, surgical process, critically ill obesity, sepsis, and trauma [1]. Clinical nutrition can play a key role in ameliorating and managing patients’ morbidities by supplying micro- and macronutrients, antioxidants, and anti-inflammatory agents [2]. Following admittance to the ICU for even a short time, malnutrition is probable, because of receiving a lack of nutritional requirements, high levels of catabolism, and physical inactivity, among other things [3]. Patients in the ICU are monitored for metabolic responses continuously, and any changes in these variables can demarcate the deterioration of illness [4]. For example, albumin (Alb) can be a marker of disease severity, but not a malnutrition indicator, whilst low levels of Alb can be associated with an increased mortality rate in hospitalized patients [5]. In addition, elevated serum lactate in critical patients is correlated with mortality rate and poor outcomes, especially in septic patients [6].

l-Carnitine is an indispensable factor in the energy production of cells that are synthesized from lysine and methionine. About one-quarter of l-carnitine is provided endogenously and three-quarters must be provided exogenously. l-Carnitine in the energy production cycle acts as a regulating agent for acetyl coenzyme A (CoA) to CoA ratio. Excess acetyl CoA can lead to malfunction, energy imbalance, and eventually malnutrition [7, 8]. It is estimated that about 300 mg/kg of l-carnitine exists in our body, predominantly in the muscles and the liver. Exogenous l-carnitine is obtained from some animal foods, where its bioavailability from foods is about 54–87% vs. 5–18% from pharmaceutical sources [9]. Therefore, mega pharmacological doses are needed in patients that cannot receive normal forms of foods orally, such as patients in ICU. On the other hand, l-carnitine deficiency is a common problem in critically ill patients because of acute kidney injury (AKI), malnutrition, prolonged parental nutrition (PN), and some infectious diseases, including human immunodeficiency virus (HIV), sepsis, trauma, and drug therapy [10]. A meta-analysis of 13 indicated that l-carnitine supplementation can significantly reduce inflammatory biomarkers (C-reactive protein (CRP), interleukin-6 (IL-6), and tumor necrosis factor-α (TNF-α)). Further subgroup analysis showed that supplementation for more than 12 weeks is more effective for CRP and TNF-α and that l-carnitine only at dosages ≥ 2 g/day can significantly decrease TNF-α [11]. Another systematic review showed that serum carnitine has an inverse association with sequential organ failure assessment score (SOFA) in septic patients, and l-carnitine supplementation leads to a reduction in the 28-mortality rate [12]. To our knowledge, limited randomized clinical controlled trials have been conducted on ICU patients about the evaluation of the effects of l-carnitine supplementation on monitoring laboratory variables. Therefore, we sought to assess the effects of a high dose of l-carnitine supplementation (3 g/day) on patients’ clinical and monitoring parameters admitted to the ICU.

## Materials and methods

### Trial design

This original prospective 1-week double-blinded parallel randomized controlled trial is a part of a whole program, in which a total of 54 patients were randomly assigned into intervention or placebo arms. Recruitment of participants was carried out between June 2018 and August 2019. The whole protocol was approved by the Research Ethics Committee of Mashhad University of Medical Sciences (registration code: IR.MUMS.fm.REC.1396.671) and was registered in the Iranian Registry of Clinical Trials (registration code: IRCT 20151108024938N2).

### Participants

Critically ill patients who were admitted to the general ICUs of Imam Reza Hospital, Mashhad, Iran, participated in this study. Our inclusion and exclusion criteria are detailed in Table 1. After checking participant eligibility, written informed consent was obtained from patients or legal guardians.

**Table 1:** Inclusion and exclusion criteria of the patients

Eligibility criteria			
Inclusion criteria	Critically illness	Exclusion criteria	Renal or liver disease
	Age > 18 y		Dialysis
	Estimated that stay in ICU longer than 2–3 days		Chemotherapy
	Patients with underlying diseases: CVD, CNS, COPD, gastrointestinal diseases, diabetes, stroke, and cancer		Pregnancy or lactation
			Any nutritional supplement use

*ICU* intensive care unit, *CVD* cardiovascular diseases, *CNS* central nervous system, *COPD* chronic obstructive pulmonary disease

### Interventions

At first, before allocation, the principal investigator (FY) explained the aims of this project to eligible participants who were admitted to the ICU and have been hemodynamically stable and received supplemental nutrition (first 24–48 h) via enteral tube feeding with 25 kcal/kg/day energy and obtained written informed consent. Nutritional support was administered in the bolus method, 7 times a day. Then, patients in the intervention group received 3 doses of 1 g l-carnitine (in total 3 g/day) in soluble form (from BSK, Zist Takhmir Co, Tehran, Iran), along with enteral feeding, for 7 days. Participants in the control group received a placebo (distilled water) with the same dose and duration.

Participants in different treatment arms received all common medications and the standard treatments, and our intervention was used as adjunctive therapy. l-Carnitine and placebo vials were produced by the BSK, Zist Takhmir Co, Tehran, Iran, with similar shape, odor, taste, and package and tagged 1 and 2.

All of the participants were visited routinely on all intervention days and probed for any undesirable effects by a physician and reported. Return empty drug vials were used for assessing adherence.

### Randomization

The block randomization method with a fixed block size of 4 was used for assigning participants into intervention or control arms. Allocation sequence generation was conducted by a blinded statistician.

### Blinding

Drugs (l-carnitine and placebo) were produced by the mentioned company with 1 or 2 labels in a similar vial package, so patients or their guardians, physicians, and investigators were fully blinded. A sealed and opaque envelope was prepared by the company and presented along with drugs that were only revealed after analysis of data to determine the contents of the vial. Furthermore, nurses, laboratory staff, and analysts were also blinded. The data coordinator and trial steering committee had access to group allocations by accessing concealment sealed envelopes.

### Outcomes

Fasting blood glucose (FBG), sodium (Na), potassium (K), calcium (Ca), magnesium (Mg), aspartate aminotransferase (AST), alanine aminotransferase (ALT), Alb, total protein, and lactate are the primary outcomes of the current study. Secondary outcomes include white blood cells (WBC), hemoglobin (Hb), platelet (PLT), blood urea nitrogen (BUN), serum creatinine (Cr), and international normalized ratio (INR). All outcomes were assessed at baseline and the 7th day after the intervention. Blood samples were drawn from patients, and after adding EDTA to samples and centrifuging at 3000 rpm and 4 °C, samples were stored at −80 °C.

All measurements were routinely performed in the central clinical laboratory of Imam Reza Hospital, Mashhad, Iran, using standard kits.

### Statistical methods

Statistical Package for the Social Sciences **(**SPSS) version 16 was used for analyzing data. Statistical significance was accepted, a priori, at a *P*-value < 0.05. Data were tested for normality by the Q-Q plot and Kolmogorov-Smirnov test. Baseline data of each group were analyzed by the chi-squared test and independent sample *t*-test. Intergroup comparisons of pre-intervention and post-intervention were assessed using a paired samples *t*-test. After adjusting baseline variables, intragroup comparisons were carried out using analysis of covariance (ANCOVA). The Muchly test of sphericity was evaluated, and when it was violated, we adopted a multivariate analysis of variance. Differences between qualitative outcomes were compared by using the chi-squared test or Fisher exact test. Continuous and categorical variables were reported as mean ± standard deviation (SD) (except for mean change of variables that were reported as mean ± standard error (SE)) and frequency (percentage), respectively. Before analysis, non-normal, positively skewed data were subjected to logarithmic transformation.

## Results

**Fig. 1:**
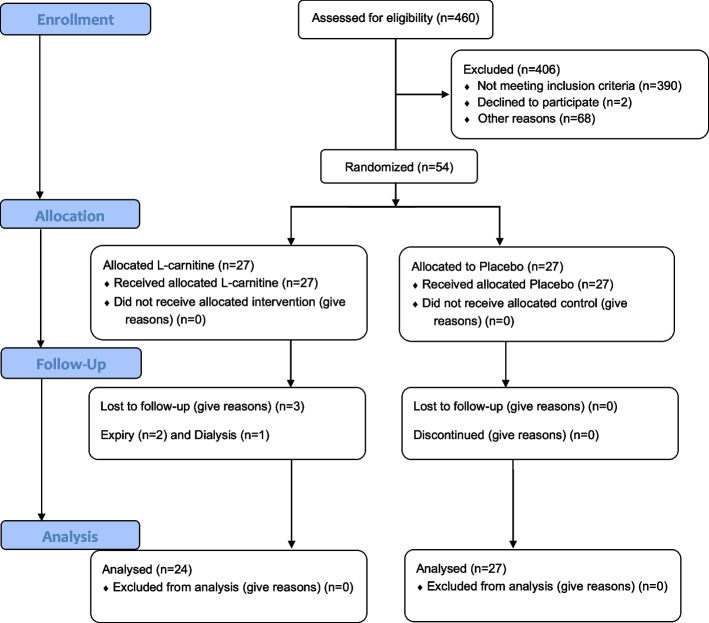
Study flow diagram

A total of 460 patients were assessed for eligibility; among them, 390 patients were excluded for not meeting the inclusion criteria (68 for personal reasons and 2 refused to take part in the study). Finally, 54 patients were randomly assigned into the intervention (3 g l-carnitine per day) and control (3 g placebo per day) groups. It is important to note that 3 participants in the intervention group dropped out (2 persons expired and 1 person for hemodialysis). In the control group, no dropout occurred. Therefore, 24 participants in the l-carnitine group and 27 in the placebo group completed the trial. The CONSORT diagram of the current study is shown in Fig. 1.

The baseline data are detailed in Table 3. The mean age of study participants was 57.7 ± 14.9 years, whilst 29 of the participants were men and 22 were women. There were no significant differences between the two groups in terms of demographic variables (Table 3) (*P*-value for all > 0.05). Differences between monitoring variables at baseline were non-significant (*P*-value > 0.05), except for total and direct bilirubin (*P*-value < 0.05). At baseline, the underlying diseases of included patients were not significantly different (all *P*-value > 0.05) (Tables 2 and 3).

**Table 2:** Participants’ underlying disease

Primary diagnosis	l-Carnitine group (*n*=24)	Control group (*n*=27)	*P*-value^1^
Respiratory diseases	12 (50)	8 (29.6)	0.13
Hypertension	10 (41.7)	13 (48.1)	0.64
Cardiovascular diseases	10 (41.7)	17 (63)	0.128
Central nervous system problems	8 (33.3)	9 (33.3)	>0.99
Diabetes	7 (29.2)	7 (25.9)	0.79
Stroke	5 (20.8)	8 (29.6)	0.47
Cancer	4 (16.7)	3 (11.1)	0.56
Gastrointestinal disease	3 (12.5)	3 (11.1)	0.87

^1^Chi-squared test

**Table 3:** Comparison of baseline characteristics between the two groups

Parameters		l-Carnitine group (*n*=24) Data: mean (SD)	Control group (*n*=27) Data: mean (SD)	*P-*value^1^
Age (years)		57.46 ± 14.44	58.22 ± 15.41	0.85
Sex	Men	14	15	0.842
	Women	10	12	
Body weight (kg) (IBW)		67.91 (10.07)	65.18 (16.08)	0.47
Energy received (kcal)		898.33 (233.23)	871.11 (237.24)	0.68
Protein (g)		56.14 (14.57)	54.44 (14.82)	0.68
Carbohydrate (g)		91.38 (23.78)	88.54 (24.28)	0.67
Fat (g)		36.49 (9.08)	35.42 (9.25)	0.68
SOFA score		8.50 (2.43)	8.25 (2.21)	0.71
NUTRIC score		5.66 (1.60)	6.11 (1.60)	0.32
WBC		11.54 (10.69)	10.14 (4.53)	0.354
Hb		10.18 (2.39)	9.60 (1.69)	0.321
PLT		219.54 (142.56)	203.74 (91.10)	0.636
PT		15.40 (5.75)	15.30 (4.55)	0.943
PTT		32.87 (6.98)	46.29 (43.46)	0.125
INR		1.44 (0.72)	1.39 (0.54)	0.808
FBG		141.79 (64.53)	122.85 (59.11)	0.279
BUN		70.58 (63.80)	66.26 (39.18)	0.769
Cr		1.37 (0.83)	1.53 (0.76)	0.467
Na		139.29 (9.59)	137.56 (7.71)	0.477
K		3.97 (0.48)	3.77 (0.60)	0.198
Ca		8.33 (0.89)	8.59 (0.50)	0.229
Mg		2.09 (0.34)	2.19 (0.60)	0.493
PH		3.30 (1.44)	3.43 (1.15)	0.722
ALT (SGPT)		41.42 (37.65)	53.53 (68.13)	0.444
AST (SGOT)		52.88 (46.50)	55.54 (59.06)	0.860
Total bilirubin		1.24 (0.81)	0.63 (0.21)	0.001
Direct bilirubin		0.57 (0.45)	0.29 (0.13)	0.007
Lactate		12.93 (6.28)	10.71 (4.38)	0.146
Alb		3.05 (0.40)	3.12 (0.48)	0.608
Total protein		5.11 (0.74)	5.16 (1.07)	0.858

^1^Resulted from independent samples *t*-test for continuous and chi-squared test for categorical variables

The mean levels of monitoring laboratory variables before and after intervention are shown in Table 4.

**Table 4:** Monitoring laboratory variables in the study groups at baseline and end of the trial

Variable	Intervention (*n*=24)				Placebo (*n*=27)				*P*-value*
	Baseline mean (SD)	End of the trial mean (SD)	*P*-value	Mean difference (SE^a^)	Baseline	End of trial	*P*-value^#^	Mean difference ± SE	
**WBC**	11.54 (10.69)	10.69 (4.90)	0.599	−0.85 (7.84)	10.14 (4.53)	11.50 (5.48)	0.191	1.36 (5.24)	0.450
Hb	10.18 (2.39)	10.00 (2.28)	0.635	−0.18 (0.36)	9.60 (1.69)	9.42 (1.82)	0.539	−0.18 (0.29)	0.660
PLT	219.54 (142.56)	239.88 (101.49)	0.539	20.34 (32.57)	203.74 (91.10)	256.92 (175.1)	0.076	53.18 (28.76)	0.551
PT	15.40 (5.75)	14.43 (4.00)	0.323	−0.97 (0.96)	15.30 (4.55)	14.84 (4.46)	0.680	−0.45 (1.07)	0.685
PTT	32.87 (6.98)	32.30 (7.66)	0.753	−0.57 (1.78)	46.29 (43.46)	35.47 (12.55)	0.222	−10.83 (8.66)	0.332
INR	1.44 (0.72)	1.29 (0.44)	0.248	−0.14 (0.12)	1.39 (0.54)	1.55 (0.83)	0.101	0.16 (0.10)	0.049
FBG	141.79 (64.53)	113.13 (56.38)	0.072	−28.67 (15.20)	122.85 (59.11)	137.11 (75.55)	0.358	14.26 (15.23)	0.128
BUN	70.58 (63.80)	55.13 (52.60)	0.187	−15.46 (11.38)	66.26 (39.18)	61.11 (41.80)	0.569	−5.1 (8.93)	0.508
Cr	1.37 (0.83)	1.00 (0.37)	0.028	−0.36 (0.16)	1.53 (0.76)	1.69 (1.07)	0.345	0.16 (0.17)	0.005
Na	139.29 (9.59)	136.33 (3.76)	0.113	−2.96 (1.79)	137.56 (7.71)	136.33 (4.51)	0.428	−1.22 (1.52)	0.815
K	3.97 (0.48)	3.85 (0.51)	0.465	−0.12 (0.16)	3.77 (0.60)	5.36 (7.38)	0.272	1.59 (1.42)	0.305
Ca	8.33 (0.89)	8.56 (1.03)	0.270	0.23 (0.20)	8.59 (0.50)	8.18 (1.06)	0.031	−0.4 (0.18)	0.044
Mg	2.09 (0.34)	2.14 (0.54)	0.694	0.05 (0.13)	2.19 (0.60)	2.16 (0.60)	0.882	−0.02 (0.16)	0.917
PH	3.30 (1.44)	3.43 (1.06)	0.654	0.14 (0.30)	3.43 (1.15)	3.93 (1.78)	0.181	0.51 (0.37)	0.263
ALT (SGPT)	41.42 (37.65)	19.88 (16.34)	0.003	−21.54 (6.55)	53.53 (68.13)	49.19 (55.30)	0.735	−4.3 (12.67)	0.022
AST (SGOT)	52.88 (46.50)	31.75 (30.20)	0.020	−21.13 (8.46)	55.54 (59.06)	44.74 (29.07)	0.349	−10.80 (11.33)	0.119
Total bilirubin	1.24 (0.81)	0.87 (0.60)	0.026	−0.37 (0.15)	0.63 (0.21)	0.69 (0.49)	0.512	0.06 (0.09)	0.833
Direct bilirubin	0.57 (0.45)	0.45 (0.35)	0.092	−0.12 (0.07)	0.29 (0.13)	0.33 (0.30)	0.494	0.04 (0.06)	0.868
Lactate	12.93 (6.28)	8.96 (6.95)	0.013	−3.97 (1.48)	10.71 (4.38)	15.72 (6.66)	0.001	5.01 (1.33)	<0.001
Alb	3.05 (0.40)	3.71 (0.62)	<0.001	0.66 (0.14)	3.12 (0.48)	3.13 (0.66)	0.912	0.01 (0.13)	0.001
Total protein	5.11 (0.74)	5.74 (0.69)	0.001	0.63 (0.16)	5.16 (1.07)	5.14 (1.05)	0.904	−0.02 (0.15)	0.003

^a^Standard error

^#^Obtained from paired *T*-test

*Obtained from analysis of covariance (ANCOVA), the mean values of outcomes were compared between groups, and adjustment was made for baseline values of compared outcomes

In regard to primary outcomes, l-carnitine supplementation significantly reduced ALT (*P*-value: 0.003), AST (*P*-value: 0.020), total bilirubin (*P*-value: 0.026), and lactate (*P*-value: 0.013) and significantly increased Alb (*P*-value: 0.000) and total protein (*P*-value: 0.001) in the intervention group, at the end of the study, compared with baseline. The between-group analysis revealed that l-carnitine significantly decreased ALT (*P*-value: 0.022), lactate (*P*-value < 0.001), and increased Ca (*P*-value: 0.044), Alb (*P*-value: 0.001), and total protein (*P*-value: 0.003) in the intervention group, in comparison with the control group (Table 4).

In regard to ancillary outcomes, l-carnitine supplementation significantly reduced Cr (*P*-value: 0.028) in the intervention group, at the end of the study, compared with baseline. The between-group analysis revealed that l-carnitine significantly decreased INR (*P*-value: 0.049) and Cr (*P*-value: 0.005) in the intervention group, in comparison with the control group (Table 4).

## Discussion

Our clinical trial, which was carried out in critically ill patients, demonstrated a statistically significant reduction in INR, Cr, ALT, and lactate and significant increases in Ca, Alb, and total protein, following l-carnitine supplementation, in comparison with the placebo group.

l-Carnitine supplementation in critically ill patients is a contemporary topic of debate [13]. To our knowledge, this is the first study to have evaluated the effects of 3 g/day l-carnitine enteral supplementation on monitoring variables in general ICU patients. In recent years, many studies have investigated l-carnitine effects on various conditions; in 2014, Lee et al. showed that 1 g/day of l-carnitine capsules resulted in a reduction of inflammation in coronary artery disease (CAD) patients [14]. A recent trial that we conducted on 56 ICU-admitted patients with 3 g l-carnitine supplements revealed that CRP and IL-6 were reduced significantly in the intervention group vs. the control group. SOFA and APACHE score which shows disease severity and clinical symptoms of the patients and predicts mortality risk were reduced significantly in the intervention group in comparison with the control group. Additionally, the duration of mechanical ventilation was reached in a significant reduction, and also, ICU discharge was significantly shorter in the l-carnitine group in comparison with the control group [15]. A meta-analysis by Song et al. which was conducted on 17 randomized clinical trials cleared that l-carnitine supplementation can improve clinical symptoms and cardiac function [16]. In another placebo-controlled trial conducted in hemodialysis patients, participants received 1 g l-carnitine for 3 months, and at the end of the trial, CRP was significantly reduced, although Alb, parathyroid hormone (PTH), and ferritin did not change [17]. Mahmoodpoor et al. administered 2 g l-carnitine per day by enteral tube feeding, in comparison with water as a placebo, to traumatic brain injury (TBI) patients for 7 days and revealed that l-carnitine can improve neurobehavioral function and cerebral edema [18]. In a randomized controlled trial carried out on 250 septic shock patients, single infusion doses of 18 g, 12 g, and 6 g of l-carnitine, vs. a saline placebo, had no effects on the 28-mortality rate at any dose [19]. Chronic critically ill ICU patients are at risk for l-carnitine deficiency due to prolonged parenteral nutrition, malnutrition, and renal failure; in addition, studies have revealed that serum carnitine tends to be notably reduced in these patients [7, 10, 20].

The findings of the current study indicated that 3 g/day l-carnitine for 7 days can increase Alb and total protein. A randomized controlled trial conducted on 42 hemodialysis patients who were supplemented with intravenous (IV) l-carnitine, at a dosage of 20 mg/kg after each hemodialysis round (three times per week), revealed that, after 6 months, total protein and Alb significantly increased in the intervention group [21]. Another trial conducted on hemodialysis patients who received 15 mg/kg of l-carnitine, intravenously, 3 times/week, found that after 3 months, total protein was increased, and after 6 months, Alb was significantly increased [22]. Nevertheless, 2000 mg/day l-carnitine or placebo oral supplementation in 46 chronic hemodialysis patients for 3 months showed no effect on serum Alb [17]. In malnourished ICU stay patients, Alb and total protein are usually decreased [23]. The Alb enhancement in the current trial might be attributable to the reduction in inflammation because it is a negative acute phase protein indicator [24]; indeed, we hypothesized that total protein increments might be due to metabolism improvement and boosting anabolism of patients. Moreover, in the current study, Ca increased significantly in the intervention group compared with the control group, which might be due to the improvement in energy hemostasis and nutritional status of the patients in response to the l-carnitine supplementation.

ALT and AST were significantly reduced in our study at day 7 in the intervention group but were only significant between the groups for ALT. Because of tissue damage, ALT and AST increased, and, typically, ALT will be raised to a greater extent than AST [25]. Concordant with our results, a randomized clinical trial that evaluated the effects of 750 mg l-carnitine 3 times per day or placebo, in 60 type 2 diabetic patients with nonalcoholic fatty liver disease (NAFLD), reported that ALT and AST declined significantly in the intervention vs. the control group [26]. In another study, 2000 mg/day l-carnitine supplementation in 74 participants with nonalcoholic steatohepatitis (NASH), for 6 months, yielded a reduction in ALT and AST in the intervention group at study cessation [27]. Interestingly, a systematic review and meta-analysis of 19 randomized trials reported a reduction of ALT and AST following l-carnitine supplementation. Moreover, in the ensuant subgroup analysis, the authors showed that l-carnitine supplementation had a significant and beneficial effect on ALT and AST, only at dosages ≥2 g/day, whilst treatment periods less than 12 weeks or more than 12 weeks were both meaningful [28]. It is plausible that a reduction in inflammatory biomarkers and hepatic fibrogenesis may have contributed to a reduction in ALT and AST.

Creatinine is the product of muscle creatine catabolism, and in the present study, it decreased significantly, which may be attributable to improved muscle damage. Similarly, in a previous meta-analysis study, l-carnitine supplementation improved muscle damage, where the authors posited that the reduction in muscle damage might be because of a concurrent improvement in oxidative stress status [29].

In accordance with our results, Hajihashemi et al. found a reduction in lactate in migraine patients in response to 8 weeks of supplementation with l-carnitine and CoQ10 (500 mg and 30 mg per day, respectively), in comparison with placebo [30]. Bakker et al. noted that decreased tissue oxygenation can increase serum lactate, which is an important practical monitoring variable in many diseases, including critically ill patients. Critically ill patients are under consistent biological stress conditions, wherein lactate will be derived from anaerobic glucose metabolism (glycolysis), which produces energy 2−3 times faster than oxidative phosphorylation, and is vital to critical patients who are in an energy crisis [31]. Once a patient enters the recovery phase, lactate converts to pyruvate for energy production in large amounts, and l-carnitine might be boosting this action.

The antioxidant and anti-inflammatory effects of l-carnitine have been discussed previously, but the concurrent effects on monitoring variables have not been reported. Our study was the first in this regard. Other strengths of our trial include the acquisition of full monitoring data. However, despite the novelty of the current study, some limitations should be noted: for instance, the low quantity of participants. Moreover, due to budgetary limitations, we were unable to assess plasma carnitine levels, which would have represented a useful addition. Heterogenous participants can be another limitation; however, given the nature of the population, this may be unavoidable. Additionally, we could not assess micronutrients in the blood, whilst precise weight and height measurements were infeasible due to the ICU conditions.

## Conclusion

Our trial results showed that l-carnitine supplementation in critically ill patients can improve several parameters, including INR, Cr, ALT, lactate, Ca, Alb, and total protein. However, more clinical trials are needed to clarify these results.

## Data Availability

The datasets that have been used for the current project are available from the corresponding author upon reasonable request.

## Abbreviations

ICU: Intensive care unit
CoA: Coenzyme A
AKI: Acute kidney injury
PN: Parental nutrition
HIV: Human immunodeficiency virus
CRP: C-reactive protein
IL-6: Interleukin-6
TNF-α: Tumor necrosis factor-α
SOFA: Sequential organ failure assessment score
FBG: Fasting blood glucose
Na: Sodium
K: Potassium
Ca: Calcium
Mg: Magnesium
AST: Aspartate aminotransferase
ALT: Alanine aminotransferase
Alb: Albumin
WBC: White blood cells
Hb: Hemoglobin
PLT: Platelet
BUN: Blood urea nitrogen
Cr: Creatinine
INR: International normalized ratio
SPSS: Statistical Package for the Social Sciences
ANCOVA: Analysis of covariance
CAD: Coronary artery disease
PTH: Parathyroid hormone
TBI: Traumatic brain injury
IV: Intravenous
NAFLD: Non-alcoholic fatty liver disease
NASH: Nonalcoholic steatohepatitis
